# Metformin increases glucose uptake and acts renoprotectively by reducing SHIP2 activity

**DOI:** 10.1096/fj.201800529RR

**Published:** 2018-10-15

**Authors:** Zydrune Polianskyte-Prause, Tuomas A. Tolvanen, Sonja Lindfors, Vincent Dumont, Mervi Van, Hong Wang, Surjya N. Dash, Mika Berg, Jette-Britt Naams, Laura C. Hautala, Harry Nisen, Tuomas Mirtti, Per-Henrik Groop, Kristiina Wähälä, Jukka Tienari, Sanna Lehtonen

**Affiliations:** * Department of Pathology, University of Helsinki, Helsinki, Finland;; † Department of Chemistry, University of Helsinki, Helsinki, Finland;; ‡ Department of Urology, Helsinki University Hospital, Helsinki, Finland;; § Folkhälsan Research Center, Folkhälsan Institute of Genetics, University of Helsinki, Helsinki, Finland;; ¶ Abdominal Center Nephrology, University of Helsinki and Helsinki University Hospital, Helsinki, Finland;; ‖ Research Program Unit, Diabetes and Obesity, University of Helsinki, Helsinki, Finland;; # Central Clinical School, Monash University, Melbourne, Victoria, Australia; and; ** Helsinki University Hospital, Hyvinkää, Finland

**Keywords:** diabetic kidney disease, insulin resistance, lipid phosphatase, podocyte, type 2 diabetes

## Abstract

Metformin, the first-line drug to treat type 2 diabetes (T2D), inhibits mitochondrial glycerolphosphate dehydrogenase in the liver to suppress gluconeogenesis. However, the direct target and the underlying mechanisms by which metformin increases glucose uptake in peripheral tissues remain uncharacterized. Lipid phosphatase Src homology 2 domain-containing inositol-5-phosphatase 2 (SHIP2) is upregulated in diabetic rodent models and suppresses insulin signaling by reducing Akt activation, leading to insulin resistance and diminished glucose uptake. Here, we demonstrate that metformin directly binds to and reduces the catalytic activity of the recombinant SHIP2 phosphatase domain *in vitro*. Metformin inhibits SHIP2 in cultured cells and in skeletal muscle and kidney of db/db mice. In SHIP2-overexpressing myotubes, metformin ameliorates reduced glucose uptake by slowing down glucose transporter 4 endocytosis. SHIP2 overexpression reduces Akt activity and enhances podocyte apoptosis, and both are restored to normal levels by metformin. SHIP2 activity is elevated in glomeruli of patients with T2D receiving nonmetformin medication, but not in patients receiving metformin, compared with people without diabetes. Furthermore, podocyte loss in kidneys of metformin-treated T2D patients is reduced compared with patients receiving nonmetformin medication. Our data unravel a novel molecular mechanism by which metformin enhances glucose uptake and acts renoprotectively by reducing SHIP2 activity.—Polianskyte-Prause, Z., Tolvanen, T. A., Lindfors, S., Dumont, V., Van, M., Wang, H., Dash, S. N., Berg, M., Naams, J.-B., Hautala, L. C., Nisen, H., Mirtti, T., Groop, P.-H., Wähälä, K., Tienari, J., Lehtonen, S. Metformin increases glucose uptake and acts renoprotectively by reducing SHIP2 activity.

Metformin is recommended as a first-line therapy for type 2 diabetes (T2D) (1). Metformin exerts its glucose-lowering effect by suppressing gluconeogenesis in the liver and facilitating glucose uptake and use by peripheral tissues (2, 3). The proposed mechanisms of action of metformin in the liver include inhibition of mitochondrial respiratory chain complex I, regulation of gene transcription *via* peroxisome proliferator-activated receptor γ coactivator 1-α, and modulation of the cellular redox state by inhibiting mitochondrial glycerolphosphate dehydrogenase (mtGPD), leading to reduced gluconeogenesis (4–6). Metformin activates energy sensor AMPK and inhibits its downstream effector mammalian target of rapamycin, but it also acts independently of AMPK (1, 7). It has been shown that mtGPD is a direct target of metformin in the liver (6). However, the direct molecular target and the exact molecular mechanism by which metformin facilitates glucose uptake in peripheral tissues have not been described.

Decreased glucose uptake may result from suppressed insulin signaling or impaired glucose transporter (GLUT) 4 trafficking. Insulin activates PI3K, which produces phosphatidylinositol (3,4,5)-trisphosphate [PI(3,4,5)P3] from phosphatidylinositol (4,5)-*bis*phosphate [PI(4,5)P2], resulting in the activation of Akt, translocation of GLUT4 to the plasma membrane (PM), and glucose uptake. Lipid phosphatase Src homology 2 domain-containing inositol-5-phosphatase 2 (SHIP2) suppresses PI3K-mediated insulin signaling by hydrolyzing PI(3,4,5)P3 to PI(3,4)P2 (8). SHIP2 is upregulated in adipose tissue, skeletal muscle, and glomeruli of diabetic mice (9, 10). In humans, a mutation in the UTR of inositol polyphosphate phosphatase-like 1, the gene encoding SHIP2, associates with T2D, and the expression of the mutant *in vitro* increases the expression of SHIP2 (11). SHIP2 knockout mice are resistant to high-fat diet-induced obesity (12), whereas mice overexpressing SHIP2 have impaired glucose tolerance (13). These data indicate that SHIP2 is a potential drug target to treat insulin resistance. Insulin resistance is also a risk factor for diabetic kidney disease (DKD) (14), which affects 20–40% of patients with diabetes and is characterized by loss of glomerular epithelial cells (podocytes) (15). SHIP2 overexpression in podocytes reduces Akt activation and induces podocyte apoptosis (10), suggesting that SHIP2 inhibition could also protect from podocyte loss.

Several SHIP2 inhibitors are known, but these are unfavorable drug candidates as a result of their poor pharmacokinetic properties and bioavailability (16). To discover SHIP2 inhibitors with better drug-like properties, we performed *in silico* structure-based virtual screening of small molecule chemical libraries and identified metformin. Here, we demonstrate that metformin directly binds to and reduces the activity of the phosphatase domain of SHIP2, providing a molecular mechanism by which metformin enhances glucose uptake and protects against podocyte loss.

## MATERIALS AND METHODS

### *In silico* virtual screening

The active site of SHIP2 was determined using both the crystal structure (3NR8) and the computational model (O15357) of SHIP2 phosphatase domain. One cavity in both structures bound the known SHIP2 inhibitor AS1949490 (17) and PI(3,4,5)P3, matching the site determined by Mills *et al.* (18). The High Throughput Biomedicine Unit (University of Helsinki, Finland) small molecule libraries were used for *in silico* virtual screening by docking the molecules to the active site of SHIP2 in both structures using Discovery Studio 4.0 (Biovia, San Diego, CA, USA).

### Cell culture and biochemical assays

Rat L6 myoblasts (American Type Culture Collection, Manassas, VA, USA) and hepatoma cells (Fao Cells, Culture Collection; Public Health England, Salisbury, United Kingdom) were maintained according to the provider’s instructions. L6 myoblasts stably expressing hemagglutinin-GLUT4-green fluorescent protein (HA-GLUT4-GFP, referred to as L6-GLUT4) were generated, as previously described (19). Immortalized human podocytes were maintained as previously described (10). Lentiviral infection was used to overexpress or knock down transiently SHIP2 in cultured cells on d 10–12 of differentiation for 48 h (basal level) or 72 h (insulin stimulation), as previously described (10, 20). Human pLKO1-short hairpin RNA (shRNA) SHIP2 (CCACCCAAGAACAGCTTCAAT) was obtained from Functional Genomics Unit, University of Helsinki. Apoptosis was measured by flow cytometry using the Annexin V-FITC Apoptosis Kit (Becton Dickinson, Franklin Lakes, NJ, USA) and double staining with 7-aminoactinomycin D (Becton Dickinson), as previously described (10). Podocytes were lysed as previously described (21). Myotubes, hepatoma cells, and tissues were lysed and immunoblotting performed as in Tolvanen *et al.* (19) with anti-SHIP2 and anti-AMPK (Santa Cruz Biotechnology, Dallas, TX, USA); anti-phospho (p)Akt (S473), anti-pAMPK (Thr172), anti-AMPK, and anti-cleaved caspase-3 (Cell Signaling Technology, Danvers, MA, USA); anti-Pan Akt (R&D Systems, Minneapolis, MN, USA); anti-tubulin, anti-Glut4, and anti-Glut1 (MilliporeSigma, Burlington, MA, USA); and anti-actin (Abcam, Cambridge, United Kingdom). Immunofluorescence and cell-surface labeling with anti-HA.11 (Covance, Princeton, NJ, USA) were performed as in Tolvanen *et al.* (19) and Heikkila *et al.* (21) using CAS-Block (Vector Laboratories, Burlingame, CA, USA).

### Production of recombinant SHIP2 and SHIP1 phosphatase domains and SHIP2 immunoprecipitation

Recombinant His-tagged human SHIP2 and SHIP1 phosphatase domains were produced as previously described (17). Lysates from myotubes, podocytes, and hepatoma cells, treated with metformin (MilliporeSigma) at indicated concentrations for 20–24 h or from tissues of mice treated with metformin for 12 d, were precleared with protein G-Sepharose (Thermo Fisher Scientific, Waltham, MA, USA) at +4°C for 1 h and incubated with anti-SHIP2 or goat IgGs for 16–20 h at +4°C. Immunocomplexes were bound to protein G-Sepharose beads at +4°C for 2 h and washed 3 times with malachite green phosphate assay buffer {10 mM 4-(2-hydroxyethyl)-1-piperazineethanesulfonic acid (HEPES), pH 7.25, 6 mM MgCl_2_, 0.1% 3-[(3-cholamidopropyl)dimethylammonio]-1-propanesulfonate (CHAPS), 250 mM sucrose, and 0.25 mM EDTA}.

### SHIP2 activity measurement

**Figure 1:**
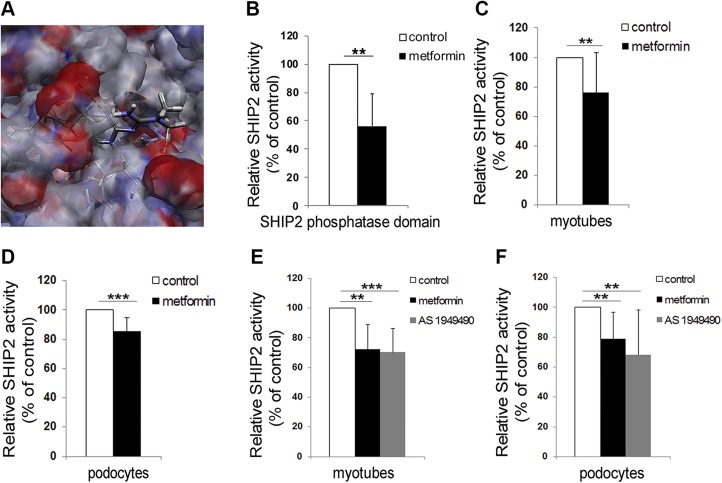
Metformin reduces the catalytic activity of SHIP2. *A*) Predicted binding orientation of metformin to the active site of SHIP2. Hydrogen bond is shown in green. Hydrophobic residues are shown in gray, positive-charged residues in blue, and negative-charged residues in red. *B*) Metformin (10 µM) reduces the activity of purified His-tagged SHIP2 phosphatase domain (100 ng). *C*, *D*) Metformin (1 mM, 20–24 h) reduces the activity of SHIP2 immunoprecipitated from the lysates of L6 myotubes (*C*) and podocytes (*D*). *E*, *F*) Metformin and AS1949490 at 20 µM concentration (20–24 h) reduce the activity of SHIP2 immunoprecipitated from the lysates of L6 myotubes (*E*) and podocytes (*F*). The catalytic activity of SHIP2 was measured by manual malachite green phosphate assay. Data are presented as the mean ± sd of 3–5 independent experiments . **P ≤ 0.01, ***P ≤ 0.001 (Student’s *t* test).

The catalytic activities of the recombinant SHIP2 and SHIP1 phosphatase domains were determined by malachite green phosphate assay at the High Throughput Biomedicine Unit (University of Helsinki) using d-myo-PI(3,4,5)P3 (Echelon Biosciences, Salt Lake City, UT, USA). Different volumes, resulting in desired final concentrations of inhibitors, were transferred to the wells of 384-well plates by using the Echo550 acoustic dispenser (Labcyte, San Jose, CA, USA). Recombinant protein (100 ng) in reaction buffer {10 mM HEPES, pH 7.25, 6 mM MgCl_2_, 0.1% CHAPS, 250 mM sucrose, and 0.25 mM EDTA} was placed in each well (7.5 µl), and the plates were preincubated at room temperature for 15 min. The phosphatase reaction was started by adding PI(3,4,5)P3 diluted in the reaction buffer (7.5 µl) to each well to reach a final concentration of 100 µM PI(3,4,5)P3 in the 15 µl reaction volume. The plates were incubated at room temperature for 20 min, after which, 15 µl BIOMOL green reagent (Biomol, Plymouth Meeting, PA, USA) was added to each well, and then the plates were incubated for another 25 min at room temperature. Absorbance was measured at 620 nm using a Pherastar FS plate reader (BMG Labtech, Offenburg, Germany). For the manual malachite green phosphate assay in 96-well plates [see **Fig. 1**; and activity measurement of phosphatase and tensin homolog (PTEN; Signalchem, Richmond, BC, Canada)], the reaction volume was increased to 50 µl, and 100 µl Biomol Green Reagent was added. The catalytic activity of SHIP2 immunoprecipitated from cell and tissue lysates was determined as in Saurus *et al.* (22), omitting SHIP2 inhibitors in lysis buffer, immunoprecipitation reaction, wash, and assay buffers. AS1949490 was synthesized, as described in Supplemental Fig. S1*A*, or purchased from Tocris Bioscience (Bristol, United Kingdom).

### Glucose uptake and GLUT4 translocation assays

Glucose uptake assay and On-Cell Western were performed essentially as in Tolvanen *et al.* (19). For HA-GLUT4-GFP endocytosis assay, L6-GLUT4 cells were incubated under basal conditions with metformin (2 mM, 20–24 h) or stimulated with insulin (100 nM, 15 min). Cells were transferred on ice; incubated with anti-HA IgG in serum-free medium for 15 min, followed by additional incubation in fresh cell differentiation medium for 15 min at +37°C; and fixed with 2% paraformaldehyde for 20 min. Cells were incubated with IRDye 800 donkey anti-mouse IgG and nuclear marker DRAQ-5 (Thermo Fisher Scientific). Detection and quantitation were performed with the Odyssey Infrared Imager (Li-Cor Biosciences, Lincoln, NE, USA).

### Metabolic assays in db/db mice

Male C57BL/Ks-db/db (BKS.Cg-m^+/+^Lepr) mice (Scanbur, Karlslunde, Denmark) were maintained according to the principles of laboratory animal care, and the experiments were approved by the National Animal Experiment Board. db/db mice (8 wk) were randomly divided into metformin and control groups (*n* = 7–8/group). Metformin (MilliporeSigma) was administered in drinking water (250 mg/kg/d) for 12 d, with the final dose administered by gavage, 4 h before euthanasia. Blood glucose concentrations and insulin tolerance test (after 12 d of metformin treatment), using human insulin (0.75 U/kg; Actrapid, Novo Nordisk, Denmark), were performed with Bayer’s Elite Glucometer after 6 h fasting. Urine (24 h) was collected individually in metabolic cages, and total urinary albumin content was measured at the Biochemical Analysis Core for Experimental Research (University of Helsinki). At the end of the experiment, mice were euthanized. Freshly dissected skeletal muscle, kidney, and liver tissues were snap frozen in liquid nitrogen, embedded in Tissue-Tek Optimal Cutting Temperature (OCT) compound (Sakura Finetek, Alphen aan den Rijn, The Netherlands), or fixed in 10% formalin, followed by embedding in paraffin. Quantitative RT-PCR was performed as in Dash *et al.* (23). The primers used to amplify phosphoenolpyruvate carboxykinase 1 (*PCK1*) were the following: forward 5′-CCAAGAGCAGAGAGACACAG-3′ and reverse 5′-CAATACCAATCTTGGCCAGC-3′ and glucose-6-phosphatase (*G6Pase*) forward 5′-CATCAATCTCCTCTGGGTGG-3′ and reverse 5′-TGCTGTAGTAGTCGGTGTCC-3′. Values were normalized to *actin*, amplified with primers forward 5′-GTTCGCCATGGATGACGATA-3′ and reverse 5′-ACATAGGAGTCCTTCTGACC-3′, or *18S RNA*, amplified with primers forward 5′-GAGGGACAAGTGGCGTTCAG-3′ and reverse 5′-ATCACGAATGGGGTTCAACG-3′.

### Immunohistochemical analysis

Kidney samples of renal cancer patients, with or without diabetes, representing nonmalignant part of the kidney, were obtained from surgical nephrectomies performed at the Helsinki and Uusimaa Hospital district. Patient characteristics are described in Supplemental Table S1. The use of human material was approved by the local Ethics Committee, and written, informed consent was received from study participants. Paraffin sections were stained with anti-SHIP2 (I-20) as in Hyvonen *et al.* (10) or anti- Wilms tumor protein 1 (WT1; Santa Cruz Biotechnology) using the Brightvision Poly-HRP-anti-Rabbit IgG kit (ImmunoLogic, Amsterdam, The Netherlands) and 3,3′-diaminobenzidine (Dako Cytomation, Carpinteria, CA, USA). Slides were counterstained with hematoxylin and digitally scanned with 3DHistech Pannoramic 250 FLASH II (3DHistech, Budapest, Hungary) at the Genome Biology Unit (University of Helsinki). Quantification of SHIP2 staining intensity was performed with the HistoQuant module (3DHistech). Podocyte number was estimated by counting the number of WT1-positive cells per glomerular cross-section in 10 glomeruli per patient in a blinded manner, as described in Guo *et al.* (24). Only glomeruli larger than 170 µm in diameter were examined to ensure sectioning through the center of each glomerulus.

### Statistical analyses

Data are shown as means ± sd or sem. Statistical analyses were performed using a 2-tailed Student’s *t* test or 1-way ANOVA with Bonferroni adjustment. Values of *P* ≤ 0.05 were considered as statistically significant.

## RESULTS

### Identification of novel SHIP2 inhibitors

To identify novel SHIP2 inhibitors *in silico*, we performed structure-based virtual screening of small molecule libraries by virtually docking the molecules to the catalytically active site of SHIP2 in both the crystal structure and the computational model of SHIP2 phosphatase domain. Screening of 88,640 molecules revealed 2636 molecules that bound to both SHIP2 structures. A total of 379 molecules had more favorable interaction energies than the previously characterized SHIP2 inhibitor AS1949490 and were selected for biologic validation. For this, we produced a His-tagged human SHIP2 phosphatase domain and tested the potency of the compounds to inhibit its catalytic activity by malachite green phosphate assay. Four compounds had half-maximal inhibitory concentration (IC_50_) values lower than 10 µM (Supplemental Table S2 and **Table 1**), and one of them was metformin (Fig. 1*A*).

**TABLE 1:** IC_50_ values and inhibition efficiencies of SHIP2 inhibitors identified in the virtual screening and their specificity for SHIP2 in relation to SHIP1 and PTEN

Molecule	SHIP2		SHIP1		PTEN	
	IC_50_ (µM)	Inhibition efficiency (%)	IC_50_ (µM)	Inhibition efficiency (%)	IC_50_ (µM)	Inhibition efficiency (%)
Metformin	6.0	39.4	>50	11.1	>50	0
Megestrol acetate	6.2	76.6	24.6	30	N/T	N/T
Mercaptopurine	8.8	52.9	21.2	11.4	N/T	N/T
Thioguanine	9	44.9	>50	29.9	N/T	N/T
AS1949490	N/A	25.9	>50	11.8	>50	0

Inhibition efficiencies were determined using the inhibitors at 100 µM concentration. The measurements for SHIP2 and SHIP1 purified His-tagged phosphatase domains were carried out in the High Throughput Biomedicine Unit. N/A, not applicable; N/T, not tested.

### Metformin inhibits SHIP2

Metformin reduces the catalytic activity of the recombinant SHIP2 phosphatase domain by 44% (Fig. 1*B*) with an IC_50_ value of 6 µM (Supplemental Fig. S1*B*). Metformin does not inhibit SHIP1, 38% homologous with SHIP2, or PTEN, a lipid 3′-phosphatase (Table 1). AS1949490 reduced the activity of the recombinant SHIP2 and SHIP1 phosphatase domains by 26 and 12%, respectively (Table 1). Enrichment of SHIP2 by immunoprecipitation from cultured cells and measurement of the phosphatase activity in the precipitate revealed that 1 mM metformin reduces SHIP2 activity by 24% in myotubes and 14% in podocytes (Fig. 1*C, D*) but not in hepatoma cells (Supplemental Fig. S2*A*). AS1949490 (10 µM) did not inhibit SHIP2 in hepatoma cells either (Supplemental Fig. S2*A*). Low, 20 µM concentration of both metformin and AS1949490 reduced SHIP2 activity by ∼30% in myotubes and 20–30% in podocytes (Fig. 1*E, F*). Metformin treatment had no effect on SHIP2 expression level in cultured cells (Supplemental Fig. S3*A–D, I, O*). These data indicate that metformin selectively and efficiently reduces SHIP2 activity in myotubes and podocytes.

### Metformin enhances glucose uptake into cells by inhibiting SHIP2 activity

**Figure 2:**
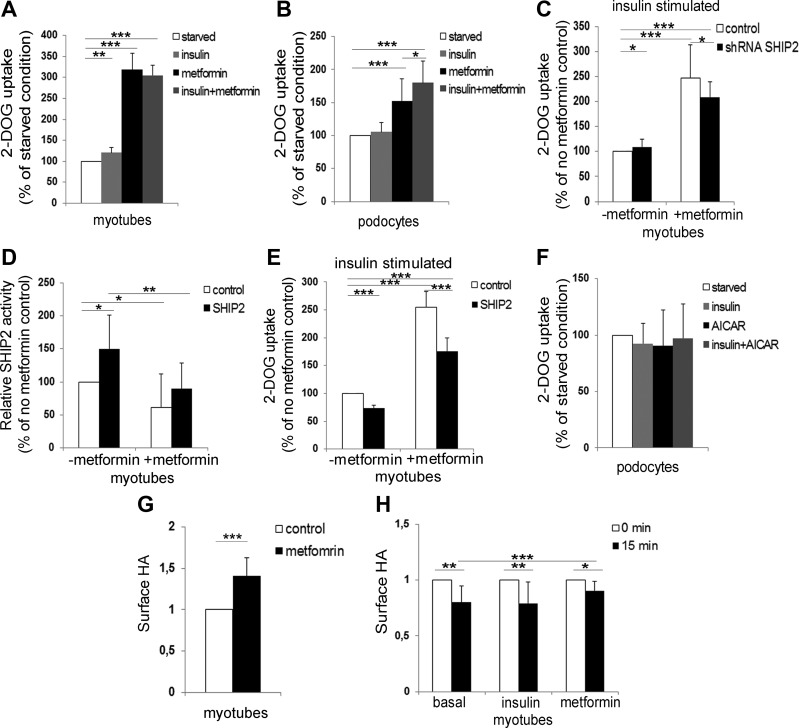
Knockdown and overexpression of SHIP2 decrease metformin-induced glucose uptake, and metformin slows down the endocytosis of GLUT4. *A*, *B*) Metformin increases glucose uptake in myotubes and podocytes. Serum-starved L6-GLUT4 myotubes (*A*) and podocytes (*B*), which were additionally starved of insulin-transferrin-selenium for 72 h, were treated with metformin (2 mM; 20–24 h) and/or stimulated with insulin (100 or 200 nM, respectively; 15 min), followed by glucose uptake assay. *C*) Knockdown of SHIP2 reduces metformin-induced glucose uptake. L6-GLUT4 myotubes were infected with SHIP2 or control shRNAs, treated with metformin (2 mM, 20–24 h) under insulin (100 nM, 15 min)-stimulated conditions, followed by glucose uptake assay. *D*) SHIP2 overexpression increases SHIP2 activity, and metformin restores it back to the level of the control. L6 myotubes overexpressing SHIP2 or empty vector (control) were incubated with metformin (1 mM; 20–24 h), and the phosphatase activity of immunoprecipitated SHIP2 was measured by malachite green phosphate assay. *E*) SHIP2 overexpression abrogates the effect of metformin to induce glucose uptake. L6-GLUT4 myotubes overexpressing SHIP2 were treated and glucose uptake performed as described in *C*. *F*) AICAR (1 mM; 20–24 h), alone or together with insulin (200 nM, 15 min), does not increase glucose uptake in podocytes. *G*) Quantification of the On-Cell Western signal for HA revealed that metformin increases the HA**-**GLUT4**-**GFP level at the PM. L6-GLUT4 myotubes were incubated under basal conditions and treated with metformin (2 mM; 20–24 h), followed by On-Cell Western assay. *H*) Metformin slows down endocytosis of HA**-**GLUT4**-**GFP in L6-GLUT4 cells. Time point 0 min indicates the amount of HA on the cell surface after metformin (2 mM; 20–24 h) or insulin (100 nM; 15 min) treatment and is set to value 1. Time point 15 min indicates the amount of HA detected on the cells after 15 min internalization. Data are presented as the mean ± sd of 3–4 independent experiments. *P ≤ 0.05, **P ≤ 0.01, ***P ≤ 0.001 (Student’s *t* test).

Insulin increased glucose uptake in L6-GLUT4 cells (Supplemental Fig. S4*A, B*) by 20% and metformin by 218%, but metformin, together with insulin, did not have an additive effect (**Fig. 2*A***). Immortalized human podocytes, cultured in medium supplemented with insulin, did not respond to insulin by increasing glucose uptake, but metformin increased glucose uptake by 52%, and metformin, together with insulin, further potentiated the effect leading to an 80% increase in glucose uptake (Fig. 2*B*).

To confirm that metformin increases glucose uptake by inhibiting SHIP2, we performed SHIP2 knockdown and overexpression experiments, both expected to reduce metformin-induced glucose uptake. Reduction of SHIP2 expression in L6-GLUT4 myotubes by 30–45% (Supplemental Fig. S5*A, B*) increased insulin-stimulated glucose uptake by 9% (Fig. 2*C*), confirming previously reported weak or no effects of SHIP2 knockout/knockdown on insulin signaling/sensitivity and glucose uptake (12, 25). Metformin enhanced glucose uptake by 148% in L6-GLUT4 myotubes, whereas metformin coupled to SHIP2 knockdown increased insulin-stimulated glucose uptake by only 109% (Fig. 2*C*), indicating that reduced expression of SHIP2 decreases the ability of metformin to enhance glucose uptake.

SHIP2 overexpression in myotubes led to an average 30–45% increase in SHIP2 expression (Supplemental Fig. S5*C, D*) and 50% increase in SHIP2 activity (Fig. 2*D*). Metformin decreased the SHIP2 overexpression-induced SHIP2 activity to the level in the control (Fig. 2*D*), and the amounts of immunoprecipitated SHIP2 used for activity measurements showed no difference between the samples confirming equal input (Supplemental Fig. S5*E*). In L6-GLUT4 myotubes, SHIP2 overexpression decreased insulin-stimulated glucose uptake by 20–25% (Fig. 2*E*). Metformin increased glucose uptake by 155% in empty vector-transfected myotubes, whereas metformin coupled to SHIP2 overexpression increased insulin-stimulated glucose uptake by only 76% (Fig. 2*E*), indicating that SHIP2 overexpression abrogates the ability of metformin to enhance glucose uptake. Collectively, these data suggest that metformin enhances glucose uptake by reducing SHIP2 activity.

### Metformin slows down endocytosis of GLUT4

SHIP2 overexpression reduces insulin-induced Akt activity (10, 13), and metformin activates AMPK (26). Metformin did not increase Akt phosphorylation (activity) in myotubes and podocytes in basal state (Supplemental Fig. S3*A, B, G, L*) but increased AMPK phosphorylation (activity) 2- to 3-fold (Supplemental Fig. 3*A–C*, *H*, *M*, *P*). A well-established pharmacological activator of AMPK, 5-aminoimidazole-4-carboxamide ribonucleoside (AICAR), increased AMPK activity 2-fold in podocytes (Supplemental Fig. S3*B, N*), but in contrast to myotubes (27), AICAR or insulin combined with AICAR failed to increase glucose uptake in podocytes (Fig. 2*F*). This suggests that AMPK activation might only partly account for the action of metformin on glucose uptake or that there may be cell type-specific differences in the action of metformin.

Metformin has been shown to increase glucose uptake in peripheral tissues by regulating GLUT trafficking (28, 29). Indeed, metformin increased the amount of HA-GLUT4-GFP on the PM by 40% in L6-GLUT4 cells (Fig. 2*G*). A 15 min HA-GLUT4-GFP uptake assay, defining endocytosis of HA-GLUT4-GFP, revealed only a 10% decrease in the amount of HA-GLUT4-GFP on the PM in metformin-treated myotubes contrary to a 20% decrease in cells at basal state or treated with insulin (Fig. 2*H*). Metformin had no effect on GLUT1 and GLUT4 protein expression levels in myotubes and podocytes (Supplemental Fig. S3*A, B, E, F, J, K*). Collectively, these data show that metformin slows down GLUT4 endocytosis without affecting its expression level.

### Metformin prevents SHIP2 overexpression-induced apoptosis in cultured podocytes

**Figure 3:**
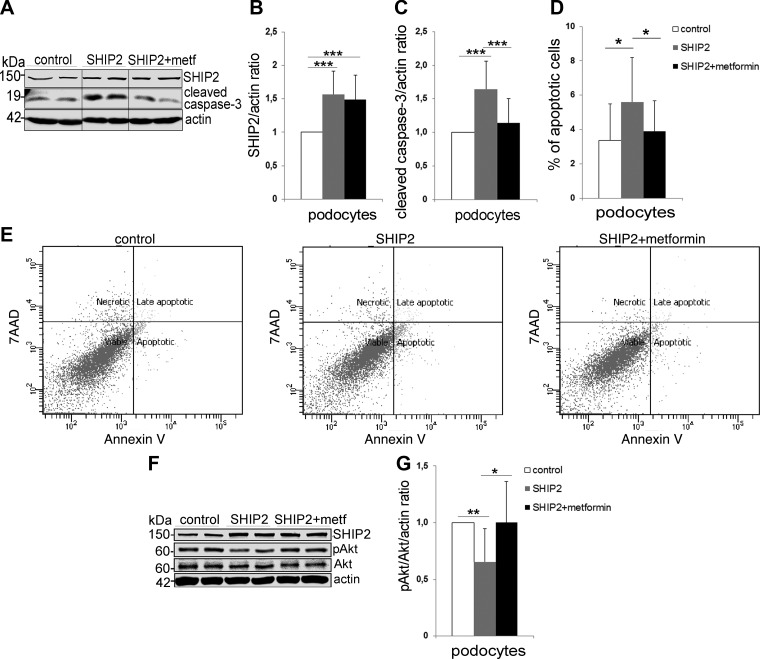
Metformin restores prosurvival Akt signaling and protects podocytes from SHIP2 overexpression-induced apoptosis. *A*) SHIP2 overexpression increases the expression of cleaved caspase-3 in podocytes, and metformin (2 mM, 48 h) restores it back to the level in the control (empty vector). Cell lysates were subjected to immunoblot analysis with anti-SHIP2, anti-cleaved caspase-3, and anti-actin IgGs. Representative protein bands are from the same immunoblot. *B*, *C*) Quantification of SHIP2 and cleaved caspase-3 levels in *A*, normalized to actin. *D*) SHIP2 overexpression increases the proportion of apoptotic cells in podocytes, and metformin (2 mM, 48 h) restores it back to the level in the control (empty vector). Apoptosis was measured by flow cytometry using Annexin V-FITC and 7-aminoactinomycin D (7AAD) double staining. *E*) Representative images of 5 independent experiments, each with 3 replicates, showing flow cytometry analysis of control (empty vector) or SHIP2-overexpressing podocytes treated or not with metformin. *F*) SHIP2 overexpression decreases insulin-induced pAkt in podocytes, and metformin restores it back to the level in the control (empty vector). Podocytes overexpressing SHIP2 or empty vector (control) were incubated with metformin (2 mM; 20–24 h) and stimulated with insulin (200 nM; 15 min). Cell lysates were subjected to immunoblot analysis with anti-SHIP2, anti-Akt, anti-pAkt, and anti-actin IgGs. *G*) Quantification of pAkt levels in *F* presented as pAkt/Akt after normalizing to actin. Data are presented as the mean ± sd of 3–5 independent experiments. *P ≤ 0.05, **P ≤ 0.01, ***P ≤ 0.001 (Student’s *t* test).

SHIP2 overexpression induces podocyte insulin resistance and apoptosis (10). SHIP2 overexpression, by 50–60% in podocytes (**Fig. 3*A, B***), increased podocyte apoptosis, as visualized by an increase in the cleavage of caspase-3, whereas metformin reduced the cleaved caspase-3 level back to the level in the control (Fig. 3*A, C*). This was further confirmed by Annexin V staining and flow cytometry analysis, showing that SHIP2 overexpression increased the level of apoptotic cells, and metformin decreased the level of apoptosis back to the level of the control (Fig. 3*D, E*). SHIP2 overexpression decreased the insulin-induced Akt phosphorylation in podocytes by 35%, whereas metformin restored its activity (Fig. 3*F, G*). These data show that metformin protects podocytes against SHIP2 overexpression-induced apoptosis by re-establishing the activity of the Akt cell-survival pathway.

### Metformin inhibits the catalytic activity of SHIP2 in muscle and kidney of db/db mice

**Figure 4:**
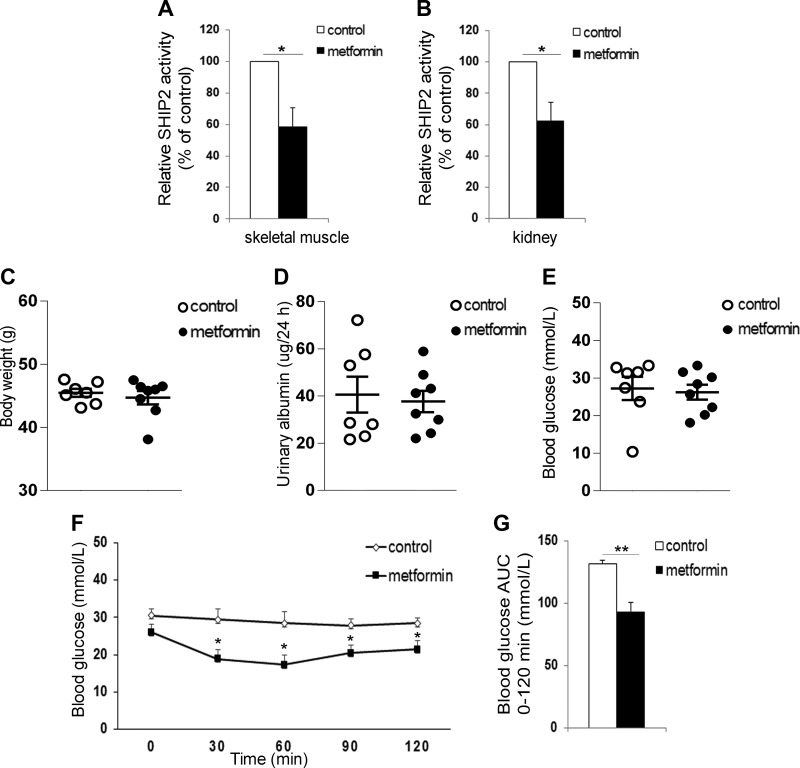
Metformin decreases the catalytic activity of SHIP2 in db/db mice. *A*, *B*) Metformin decreases the catalytic activity of SHIP2 in skeletal muscle (*A*) and kidney (*B*) of db/db mice. SHIP2 was immunoprecipitated from tissue lysates and its catalytic activity measured by malachite green phosphate assay (*n* = 6–8). *C*–*E*) Twelve d metformin treatment has no effect on body weight (*C*), urinary albumin excretion (*D*), and fasting blood glucose (*E*) in db/db mice (*n* = 7–8). *F*, *G*) Metformin improves insulin sensitivity of db/db mice. Insulin tolerance test was performed and blood glucose measured at the indicated times after insulin injection, and the area under the curve (AUC) was calculated (*n* = 5–6). Data are presented as the mean ± sem. *P ≤ 0.05, **P ≤ 0.01 (Student’s *t* test).

To confirm that metformin targets SHIP2 *in vivo*, we administered metformin daily to diabetic db/db mice at 250 mg/kg per day, which when normalized to body-surface area, translates to a human metformin dose of 20 mg/kg per day (1). Metformin treatment decreased the enzymatic activity of SHIP2 by 40% in the skeletal muscle and kidney (**Fig. 4*A, B***) without affecting SHIP2 expression level (Supplemental Fig. S6*A–D, I, N**)*. SHIP2 activity in the liver was not reduced by metformin (Supplemental Fig. S2*B*). Metformin did not activate Akt or AMPK (Supplemental Fig. S6*A–C, G, H, L, M, O*) or increase the expression levels of GLUT1 and GLUT4 in skeletal muscle or kidney (Supplemental Fig. S6*A, B, E, F, J, K*). Metformin had no effect on body weight, urinary albumin excretion, or fasting blood glucose concentrations compared with nontreated db/db mice (Fig. 4*C–E*). Insulin tolerance test, assessing whole-body glucose metabolism, revealed that metformin reduces fasting blood glucose at 30–120 min after insulin administration and the glucose area under the curve, whereas the blood glucose concentrations were unchanged in the nontreated db/db mice (Fig. 4*F, G*). In addition, metformin decreased the mRNA levels of the key gluconeogenesis genes, PCK1 and G6Pase, in liver of db/db mice (Supplemental Fig. S2*C, D*), confirming the effectiveness of our 12 d treatment. In contrast to this, metformin increased the mRNA levels of PCK1 and G6Pase in kidney of db/db mice (Supplemental Fig. S2*E, F*). These data collectively indicate that metformin reduces the activity of SHIP2 in skeletal muscle and kidney cells both *in vitro* and *in vivo* and enhances insulin sensitivity in db/db mice.

### The catalytic activity of SHIP2 and podocyte loss are decreased in kidneys of human patients with T2D receiving metformin

**Figure 5:**
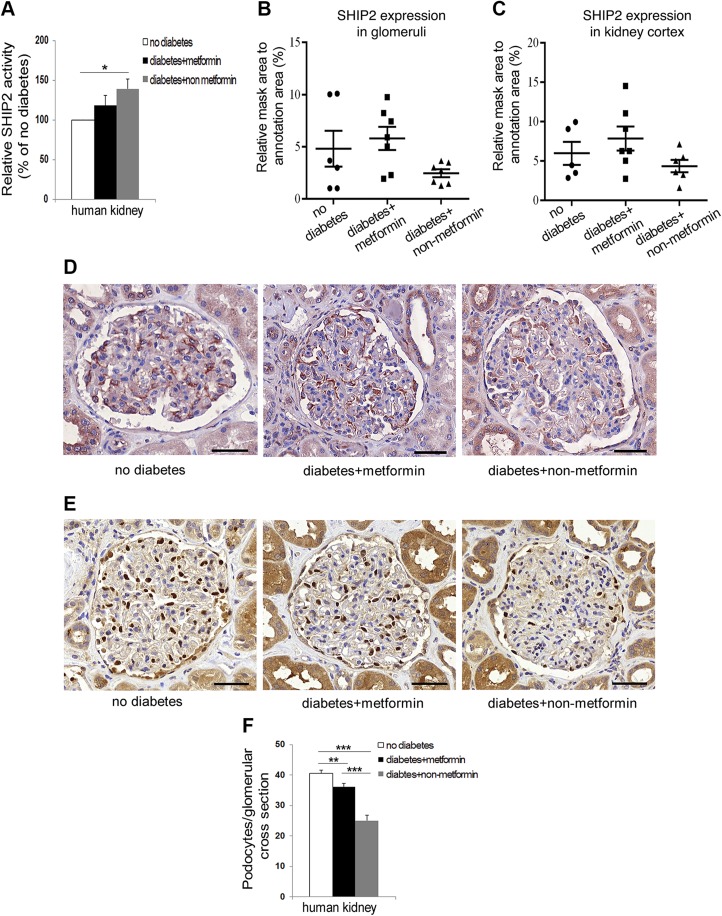
SHIP2 activity and podocyte loss are reduced in kidneys of human patients with T2D receiving metformin. *A*) SHIP2 activity is increased in kidneys of patients with T2D receiving nonmetformin medication compared with people without T2D. SHIP2 activity in patients with T2D receiving metformin medication is similar to people without T2D. Kidney samples were lysed, and the enzymatic activity of immunoprecipitated SHIP2 was analyzed by malachite green phosphate assay (*n* = 4–7). *B*) SHIP2 expression level in glomeruli in *D* was quantified with the HistoQuant (3DHistech) program. Ten randomly chosen glomeruli were analyzed from each patient (*n* = 6–7). *C*) SHIP2 expression level in kidney cortex was quantified with 3DHistech (*n* = 5–7). *D*) SHIP2 staining in human glomeruli of patients with T2D receiving metformin or nonmetformin medication and people without T2D. Paraffin sections of nephrectomy samples were processed for immunoperoxidase staining and labeled with anti-SHIP2 IgG. *E*) WT1 staining in human glomeruli of patients with T2D receiving metformin or nonmetformin medication and people without T2D. Paraffin sections of nephrectomy samples were processed for immunoperoxidase staining and labeled with anti-WT1 IgG. *F*) Podocyte loss is decreased in kidneys of patients with T2D receiving metformin medication compared with patients receiving nonmetformin medication. Podocyte number per glomeruli was quantified by counting WT1-positive cells. Ten randomly chosen glomeruli were analyzed from each patient (*n* = 9–30). Original scale bars, 50 μm. Data are presented as means ± sem. *P ≤ 0.05, **P ≤ 0.01, ***P ≤ 0.001; ANOVA (*B–F*); Student’s *t* test (*A*).

In humans, SHIP2 activity was 39% higher in the kidneys of patients with T2D without clinical nephropathy and who received nonmetformin medication (insulin or sulfonylurea), whereas the SHIP2 activity in the kidneys of patients with T2D receiving metformin was similar to that in people without diabetes (**Fig. 5*A*** and Supplemental Table S1). SHIP2 expression level showed no difference in glomeruli and kidney cortex between the groups (Fig. 5*B–D*). The number of podocytes per glomeruli was decreased by 38% in patients with T2D with nonmetformin medication, whereas metformin-receiving patients showed only 11% podocyte loss compared with people without T2D (Fig. 5*E, F*). These data on patient kidney samples suggest that metformin reduces the catalytic activity of SHIP2 and protects against podocyte loss. This, together with the data on cultured cells and db/db mice, proposes that inhibition of SHIP2 by metformin could be an important mechanism to increase glucose uptake into cells and ameliorate kidney injury in patients with T2D.

## DISCUSSION

We performed structure-based virtual screening to identify new, SHIP2-specific inhibitors, and a highlight of the screening was the identification of the anti-diabetic drug metformin. Studies have proposed various mechanisms of action for metformin to reduce gluconeogenesis in the liver (5) or *via* affecting the gut microbiota (30), reporting mainly indirect effects of metformin through various pathways involved in the metabolic control. Thus far, only Madiraju *et al.* (6) and this study have revealed tissue-specific, direct cellular targets of metformin, mtGPD in liver, and SHIP2 in muscle and kidney, contributing to the effect of metformin to reduce gluconeogenesis and increase glucose uptake, respectively.

We observed that both metformin and AS1949490 reduce the enzymatic activity of SHIP2 in cultured myotubes and podocytes but not in hepatoma cells. Also *in vivo* in db/db mice, metformin inhibited SHIP2 in the skeletal muscle and kidney but not in the liver, even though the expression of the rate-limiting enzymes of gluconeogenesis was reduced. This suggests that metformin does not suppress gluconeogenesis *via* SHIP2 inhibition in liver. Previously, SHIP2 inhibitors AS1949490 and *N*-[4-(4-chlorobenzyloxy)pyridin-2-*yl*]-2-(2,6-difluorophenyl)-acetamide were also found to reduce PCK1 expression in db/db mice (31). However, the authors did not measure whether SHIP2 activity in the liver was reduced by the treatments, leaving open whether the effect was a result of direct inhibition of SHIP2 or occurred indirectly *via* another target. The reason why metformin does not inhibit SHIP2 in the liver is unclear. It is possible that there are certain organ-specific mechanisms that determine to which target protein metformin binds. For example, the interaction partners of SHIP2 and mtGPD and the protein complexes formed thereby may affect the binding of metformin. This remains to be analyzed in future studies.

We (10) and others (9) previously showed that SHIP2 is upregulated in various tissues in rodent models of T2D. However, SHIP2 activity may be enhanced in diabetes without an increase in its expression level, as was observed in the kidneys of patients with T2D. Furthermore, decreased SHIP2 activity after metformin treatment was not a result of reduced expression of SHIP2. Collectively, our *in vitro* and *in vivo* data indicate that metformin acts on SHIP2 by reducing its activity, without an influence on its expression level.

Metformin enhances glucose uptake into cells (27, 32). We show that this occurs by reducing SHIP2 activity, as knockdown and overexpression of SHIP2 decreased the ability of metformin to induce glucose uptake in myotubes. In line with this, we observed that metformin improves insulin sensitivity in db/db mice, supporting the potential of SHIP2 as a target to improve glucose metabolism. Involvement of SHIP2 in glucose metabolism is further supported by a similar short-term AS1949490 treatment, which reduced blood glucose and improved insulin sensitivity in db/db mice (17). In line with previous literature (33, 34), we observed no difference in body weight, albuminuria, or fasting blood glucose in db/db mice after short-term metformin treatment. The latter could be explained by our finding that the renal expression of PCK1 and G6Pase was increased after metformin treatment, apparently enhancing gluconeogenesis in the kidney cortex, compensating for the reduced hepatic glucose production. This accords with the role of the kidney in gluconeogenesis (35) and is supported by a previous study revealing that 10 wk metformin treatment of db/db mice exacerbates the diabetes-induced increase in the expression of gluconeogenic genes in the kidney (34). The regulation of gluconeogenesis by metformin is complex (5). In the liver, metformin apparently does not inhibit gluconeogenic gene expression directly at the transcriptional level but inhibits gluconeogenesis *via* decreasing the hepatic energy state by reducing intracellular ATP content (36) or affecting the cellular redox state (6). The exact molecular mechanism *via* which SHIP2 inhibition in the kidney or as a compensatory response to reduced hepatic gluconeogenesis leads to an increase in renal gluconeogenesis remains to be analyzed in the future. Collectively, the data described above, together with the observation that metformin inhibits SHIP2 *in vitro* and *in vivo*, support the idea that metformin improves insulin sensitivity and facilitates glucose uptake in peripheral tissues by reducing SHIP2 activity.

In search for the mechanism by which metformin enhances glucose uptake, we analyzed the activation of both the PI3K/Akt and AMPK signaling pathways. Metformin did not activate Akt in any cell or tissue type in basal conditions (but restored SHIP2 overexpression-induced reduction in Akt activation). Metformin activated AMPK *in vitro* and showed a trend to be activated *in vivo* in liver, reflecting literature revealing both AMPK-dependent and -independent mechanisms of action for metformin (27, 32, 36). Furthermore, we found that AICAR failed to increase glucose uptake in podocytes, indicating that although metformin activates AMPK in cultured cells, this appears to make a relatively small contribution toward the overall increase in metformin-stimulated glucose uptake. Metformin may also signal *via* pathways that regulate the trafficking of GLUTs, as it affects the configuration and/or interaction of proteins at the PM, including the membrane-inserted GLUTs (37), without altering total GLUT1 and GLUT4 content (38). Indeed, we found that metformin increases GLUT4 at the PM and verified that metformin slows down GLUT4 endocytosis, whereas insulin does not, as previously observed (29, 39). This may be a result of the altered balance of phosphoinositides at the PM, as an increased level of PI(4,5)P2, also a substrate of SHIP2 (40) and expected to increase upon SHIP2 inhibition, reduces endocytosis of GLUT4 in adipocytes (41).

Insulin resistance and podocyte apoptosis contribute to the development of renal damage (42). Furthermore, a recent study proposing novel subgrouping of diabetes into 5 clusters reveals that individuals with severe insulin resistance have a high risk to develop DKD (43). We previously found that SHIP2 overexpression suppresses the Akt prosurvival signaling pathway and enhances podocyte apoptosis (10), and thus, upregulation of SHIP2 expression or activity might contribute to insulin resistance and loss of podocytes in DKD. Notably, we observed that SHIP2 activity is elevated in kidneys of patients with T2D receiving nonmetformin medication compared with people without diabetes and that metformin reduces the elevated activity of SHIP2 in the kidneys of patients with T2D, proposing a mechanism *via* which metformin acts renoprotectively. Furthermore, our *in vitro* data reveal that SHIP2 overexpression reduces insulin-induced Akt activation and that metformin restores the reduced Akt activity back to normal and protects podocytes from SHIP2 overexpression-induced apoptosis. Supporting this, we observed that podocyte loss was attenuated in metformin-treated T2D patients. This is corroborated by recent studies showing that metformin prevents renal podocyte injury in a rat model of T2D by repressing oxidative stress, reduces high-glucose-induced apoptosis in cultured podocytes *via* activation of AMPK and inhibition of mammalian target of rapamycin, and decreases albuminuria in patients with T2D (44–47). Podocyte apoptosis is an early glomerular phenotype that coincides with the onset of albuminuria and is associated with progressive podocyte depletion in DKD (15). We propose that metformin could prevent the progression of DKD at an early stage by ameliorating insulin resistance and preventing podocyte loss *via* inhibition of SHIP2 activity. This accords with Ahlqvist *et al* (43), noting that the proportion of patients with severe insulin resistance receiving metformin was low, even though this subgroup would be expected to benefit the most from metformin treatment. The finding that metformin acts on its direct cellular target, SHIP2, in human kidney tissue *in vivo* is a significant finding, as the inhibitory effect of metformin on mtGPD still awaits for confirmation in human liver.

Inhibitors identified in virtual screening included Food and Drug Administration-approved drugs used to treat cardiovascular disease, certain types of cancer, autoimmune diseases, anorexia, cachexia, and weight loss associated with cancer and AIDS (1, 48–50). Emerging evidence indicates that in addition to regulating insulin signaling, cytoskeleton remodeling, and receptor endocytosis (51), SHIP2 is implicated in the development and progression of certain types of cancer (52). This, together with our *in silico* data, confirms the importance of SHIP2 as a drug target for diabetes and specific types of cancer.

In summary, we show that metformin binds directly to the phosphatase domain of SHIP2 and reduces its activity *in vitro* and *in vivo*. Furthermore, podocyte loss was decreased in the kidneys of patients with T2D receiving metformin, suggesting a novel mechanism for the renoprotective effects of metformin. Thus, our findings unravel a novel molecular mechanism by which metformin enhances glucose transport and protects against podocyte loss.

## Supplementary Material

This article includes supplemental data. Please visit *http://www.fasebj.org* to obtain this information.

## Abbreviations

AICAR: 5-aminoimidazole-4-carboxamide ribonucleoside
DKD: diabetic kidney disease
G6Pase: glucose-6-phosphatase
GFP: green fluorescent protein
GLUT: glucose transporter
HA: hemagglutinin
IC_50_: half-maximal inhibitory concentration
mtGPD: mitochondrial glycerolphosphate dehydrogenase
PCK1: phosphoenolpyruvate carboxykinase 1
PI(3,4,5)P3: phosphatidylinositol (3,4,5)-trisphosphate
PI(4,5)P2: phosphatidylinositol (4,5)-*bis*phosphate
PM: plasma membrane
PTEN: phosphatase and tensin homolog
shRNA: short hairpin RNA
SHIP1 or 2: Src homology 2 domain-containing inositol-5-phosphatase 1 or 2
T2D: type 2 diabetes
WT1: Wilms tumor protein 1
