# Supplementary material for: Metformin increases glucose uptake and acts renoprotectively by reducing SHIP2 activity
Source: FASEB J. 2018 Oct 15;33(2):2858–69. doi: 10.1096/fj.201800529RR (PMC6338644; doi:10.1096/fj.201800529RR)

Polianskyte-Prause *et al.* Metformin increases glucose uptake and acts renoprotectively by reducing SHIP2 activity

Supplemental Table S1. Patient characteristics. Patients with type 2 diabetes did not have clinical nephropathy. Statistical analysis was performed using Student's *t*-test.

	**no diabetes**	**diabetes**	**diabetes**
n	34	27	10
Male/female	21/13	19/8	2/8
Age (years)	66±1.9	68±2.3 (p=0.57)	72±3.1 (p=0.16)
Medication		metformin (n=15) or metformin plus insulin (n=3), or metformin plus sitagliptin (n=3), or metformin plus viltagliptin (n=1), or metformin plus rosiglitazone (n=1), or metformin plus sulfonylurea (n=2), or metformin plus sulfonylurea and insulin (n=1), or metformin plus sitagliptin and rosiglitazone (n=1)	insulin (n=8) or sulfonylurea (n=2)

Supplemental Table S2. Details of potent SHIP2 inhibitors identified by virtual screening. The table comprises of the systematic (IUPAC) name, clinical uses and references.

**Molecule**	**Systematic (IUPAC) name**	**Clinical use**	**References**
Metformin	1,1-Dimethylbiguanide hydrochloride	Type 2 diabetes, cardiovascular disease, diabetic nephropathy, cancer	(1-3)
Megestrol acetate	17-(acetyloxy)-6-methylpregna-4,6-diene-3,20-dione	Treatment of breast and endometrial cancers; anorexia, cachexia, and weight loss associated with cancer and AIDS.	(4-6)
Mercaptopurine	3,7-dihydropurine-6-thione	Treatment of autoimmune diseases and acute lymphoblastic leukemia	(7, 8)
Thioguanine	2-Amino-1H-purine-6(7H)-thione	Treatment of acute myelogenous and lymphoblastic leukemias	(9, 10)

Supplemental References

1. Lalau, J. D., Arnouts, P., Sharif, A., and De Broe, M. E. (2015) Metformin and other antidiabetic agents in renal failure patients. *Kidney International* **87**, 308-322

2. Song, I. S., Han, J., and Lee, H. K. (2015) Metformin as an anticancer drug: A commentary on the metabolic determinants of cancer cell sensitivity to glucose limitation and biguanides. *Journal of Diabetes Investigation* **6**, 516-518

3. Ferrannini, E., and DeFronzo, R. A. (2015) Impact of glucose-lowering drugs on cardiovascular disease in type 2 diabetes. *European Heart Journal* **36**, 2288-2296

4. Aoyagi, T., Terracina, K. P., Raza, A., Matsubara, H., and Takabe, K. (2015) Cancer cachexia, mechanism and treatment. *World Journal of Gastrointestinal Oncology* **7**, 17-29

5. Shan, W., Wang, C., Zhang, Z., Gu, C., Ning, C., Luo, X., Zhou, Q., and Chen, X. (2014) Conservative therapy with metformin plus megestrol acetate for endometrial atypical hyperplasia. *Journal of Gynecologic Oncology* **25**, 214-220

6. Dutt, V., Gupta, S., Dabur, R., Injeti, E., and Mittal, A. (2015) Skeletal muscle atrophy: Potential therapeutic agents and their mechanisms of action. *Pharmacological Research* **99**, 86-100

7. Amin, J., Huang, B., Yoon, J., and Shih, D. Q. (2015) Update 2014: Advances to optimize 6-mercaptopurine and azathioprine to reduce toxicity and improve efficacy in the management of IBD. *Inflammatory Bowel Diseases* **21**, 445-452

8. Mei, L., Ontiveros, E. P., Griffiths, E. A., Thompson, J. E., Wang, E. S., and Wetzler, M. (2015) Pharmacogenetics predictive of response and toxicity in acute lymphoblastic leukemia therapy. *Blood Reviews* **29**, 243-249

9. Zhang, F., Fu, L., and Wang, Y. (2013) 6-thioguanine induces mitochondrial dysfunction and oxidative DNA damage in acute lymphoblastic leukemia cells. *Molecular & Cellular Proteomics : MCP* **12**, 3803-3811

10. Munshi, P. N., Lubin, M., and Bertino, J. R. (2014) 6-thioguanine: A drug with unrealized potential for cancer therapy. *The Oncologist* **19**, 760-765

Supplemental Figure legends

Supplemental Figure 1. Synthesis of 3-[(4-chlorophenyl)methoxy]-N-](S)-1-phenylethyl]-2-thiophenecarboxamide ((S)-6), (AS1949490) and inhibition of SHIP2 with metformin. *A*) 3-Hydroxy-2-thiophenecarboxylic acid methyl ester (**1**, 200 mg, 1 eq.), 4-chlorobenzylchloride (**2**, 242 mg, 1.2 eq.) and K_2CO_3 (262 mg, 1.5 eq.) were suspended in methylethylketone (MEK, 4 ml), and refluxed overnight to give 3-[(4-chlorophenyl)methoxy]-2-thiophenecarboxylic acid methyl ester **3** (quantitative yield). The ester **3** was dissolved in THF/MeOH (1:1.25, 4.5 ml) and 2N aq. NaOH (1.66 ml) was added. This solution was stirred for 4 h at 50 °C, giving the free carboxylic acid **4** (282 mg, 95%) after acidification. For amidation, the acid **4** (250 mg) was dissolved in dry DMF (3 ml), and hydroxybenzotriazole (HOBT, 145 mg, 1.15 eq.) and (*S*)-1-phenylethylamine (**(*S*)-5**, 130 μL, 1.1 eq.) and *N*-(3-dimethylaminopropyl)-*N*'-ethylcarbodiimide hydrochloride (WSCD.HCl, 205 mg, 1.15 eq.) were added. After stirring overnight at RT, flash chromatography (hexane:EtOAc 1:1) and recrystallization (abs. EtOH) gave **(*S*)-6** in 72% overall yield (mp 94 °C, m/z 371, $[\alpha]_D$ +38°, c = 1, $CHCl_3$). *B*) Dose-dependent inhibition of recombinant human SHIP2 phosphatase domain activity with metformin. The catalytic activity of SHIP2 was measured by malachite green phosphate assay at High Throughput Biomedicine Unit. Signal output is converted to percent of normalized absorbance. Each concentration point had three technical replicates.

Supplemental Figure 2. Metformin does not inhibit the catalytic activity of SHIP2 in cultured hepatoma cells and in liver of db/db mice, but, opposite to kidney, decreases the expression of gluconeogenesis genes. *A*) Metformin (1 mM) and AS1949490 (10 μM) (20-24h) do not inhibit the catalytic activity of SHIP2 immunoprecipitated from the lysates of hepatoma cells. The catalytic activity of SHIP2 was measured by malachite green phosphate assay. *B*) Metformin does not inhibit the catalytic activity of SHIP2 in liver of db/db mice. SHIP2 was immunoprecipitated from tissue lysates and its catalytic activity measured by malachite green phosphate assay (n=6-7). *C-F*) Metformin affects the expression of gluconeogenesis genes. The expression levels of the rate-limiting gluconeogenesis genes PCK1 (C and E) and G6Pase (D and F) in the liver and kidney were analyzed by quantitative RT-PCR (n=6). For quantitative RT-PCR, actin and S18 were used as internal controls for liver and kidney, respectively. The bars show the mean expression in arbitrary units. Data are presented as means ± SD of three independent experiments or as means ± SEM (*$P \le 0.05$, **$P \le 0.01$, Student's *t* test).

Supplemental Figure 3. Metformin does not affect the expression level of SHIP2, Akt and glucose transporters in myotubes and podocytes, but activates AMPK in myotubes, podocytes and hepatoma cells in culture. *A* and *B*) Metformin has no effect on SHIP2, GLUT1 and GLUT4 expression levels or Akt phosphorylation, but it activates AMPK in L6 myotubes (A) and podocytes (B). AICAR activates AMPK in podocytes (B). Cells were treated with 2 mM metformin or 1 mM AICAR for 20-24 h and lysates were subjected to immunoblot analysis with anti-SHIP2, anti-GLUT1, anti-GLUT4, anti-Akt, anti-pAkt, anti-AMPK, anti-pAMPK, anti-actin and anti-tubulin IgGs. *C*) Metformin has no effect on SHIP2 expression level, but it activates AMPK in hepatoma cells.

Treatments and immunoblotting were performed as described above. *D-P*) Quantification of SHIP2 (D, I, O), GLUT1 (E, J) and GLUT4 (F, K) levels in (A, B, C) normalized to either tubulin or actin. Quantification of pAkt (G, L) and pAMPK (H, M, N, P) levels in (A, B, C) presented as pAkt/Akt or pAMPK/AMPK after normalizing to either tubulin or actin. Data are presented as means ± SD of three-four independent experiments (**P≤0.01, ***P≤0.001, Student's *t* test).

Supplemental Figure 4. Characterization of L6-GLUT4 cells. *A*) Expression of HA-GLUT4-GFP in L6-GLUT4 cells visualized by GFP (green color). GFP was used to sort the cells with fluorescence-activated cell sorting according to their HA-GLUT4-GFP expression level to obtain homogenous populations of cells. To visualize the GFP-tag, L6-GLUT4 myoblasts were fixed with 2% paraformaldehyde. *B*) Surface labeling of L6-GLUT4 cells under basal conditions with anti-HA IgG (red color) visualizing GLUT4 at the plasma membrane. The HA-tag is located in the extracellular domain of GLUT4 and can be used to detect the insertion and exposure of GLUT4 at the cell surface. Scale bar: 50 μm.

Supplemental Figure 5. SHIP2 knockdown and overexpression in cultured myotubes by lentiviral infection. *A*) Representative immunoblot of SHIP2 knockdown efficiency in myotubes infected with shRNA construct targeting SHIP2 or empty vector shRNA (control). Cell lysates were subjected to immunoblot analysis with anti-SHIP2 and anti-tubulin IgGs. *B*) Quantification of SHIP2 expression level in (A) normalized to tubulin reveals decreased SHIP2 level in SHIP2 knockdown cells. *C*) Representative immunoblot for SHIP2 in myotubes overexpressing SHIP2 or empty vector (control). Cell lysates were subjected to immunoblot analysis with anti-SHIP2 and anti-tubulin IgGs. *D*) Quantification of SHIP2 expression level in (C) normalized to tubulin reveals increased SHIP2 level in SHIP2-overexpressing cells. *E*) Representative immunoblot for SHIP2 of SHIP2 or control IgG immunoprecipitates from L6 cells infected either with SHIP2 or empty vector (control). Infected cells were treated with metformin (2 mM, 20-24 h) and immunoblotting were performed as described above. Data are presented as means ± SD of three-five independent experiments (***P≤0.001, Student's *t* test).

Supplemental Figure 6. Metformin does not affect the expression level of SHIP2, Akt, AMPK and glucose transporters in tissues of db/db mice. *A* and *B*) Metformin has no effect on SHIP2, GLUT1 and GLUT4 expression levels, or Akt and AMPK phosphorylation in skeletal muscle (A) and kidney (B) of db/db mice. Tissue lysates from metformin-treated and control db/db mice were subjected to immunoblot analysis with anti-SHIP2, ati-GLUT1, anti-GLUT4, anti-Akt, anti-pAkt, anti-AMPK, anti-pAMPK and anti-tubulin IgGs. *C*) Metformin has no effect on SHIP2 expression level and AMPK phosphorylation in liver. Immunoblotting was performed as described above. *D-O*) Quantification of SHIP2 (D, I, N), GLUT1 (E, J) and GLUT4 (F, K) expression levels in (A, B, C) normalized to tubulin. Quantification of pAkt (G, L) and pAMPK (H, M, O) levels in (A, B, C) presented as pAkt/Akt and pAMPK/AMPK after normalizing to tubulin. Data are presented as means ± SEM (Student's *t* test).

Supplemental Figure 1

A

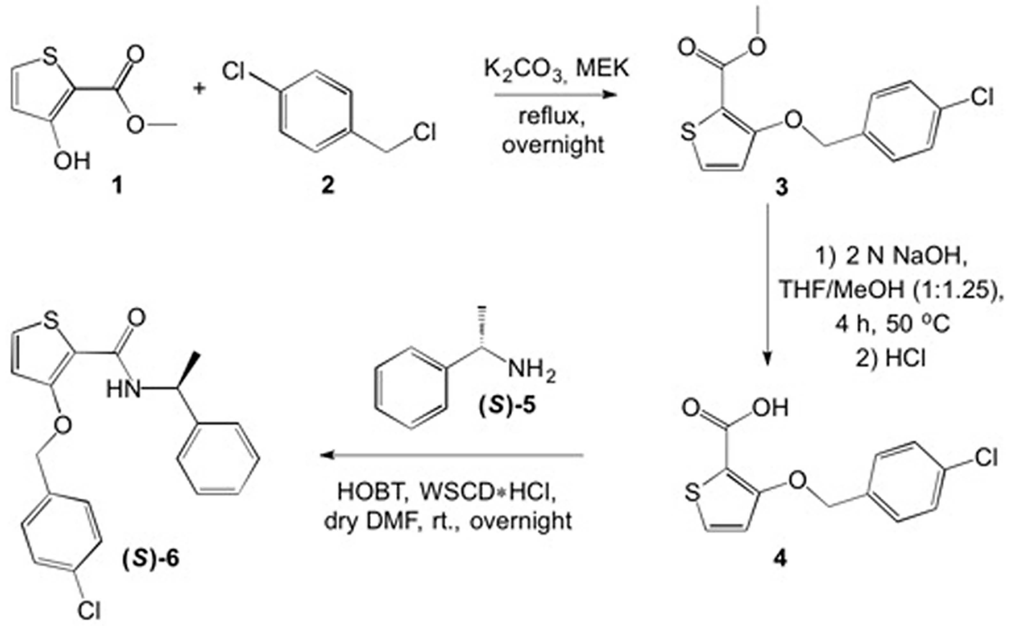

B

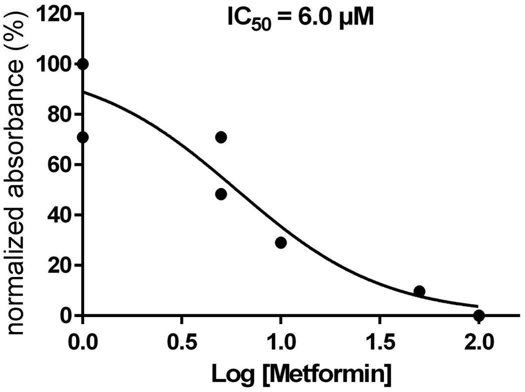

Supplemental Figure 2

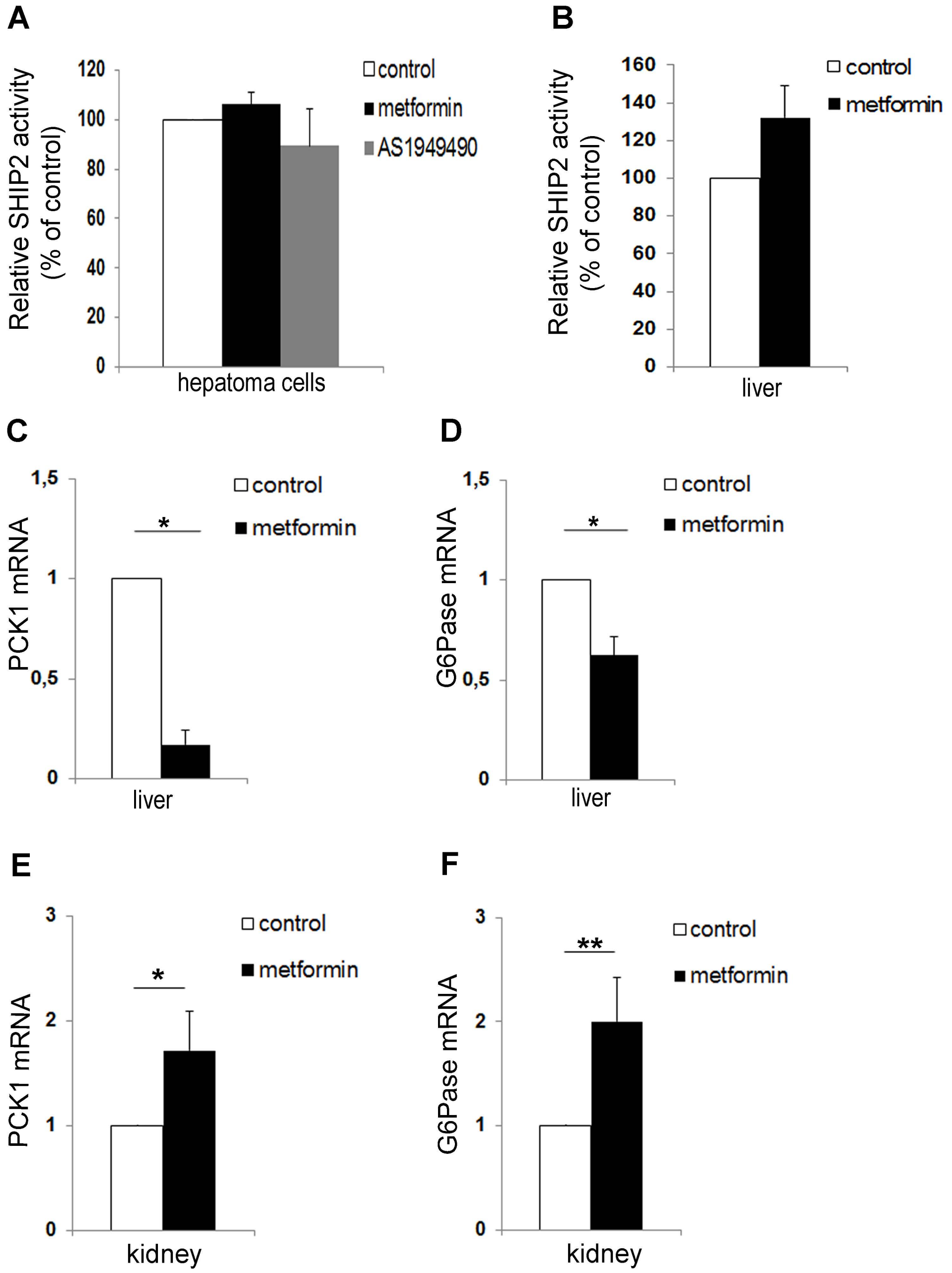

Supplemental Figure 3

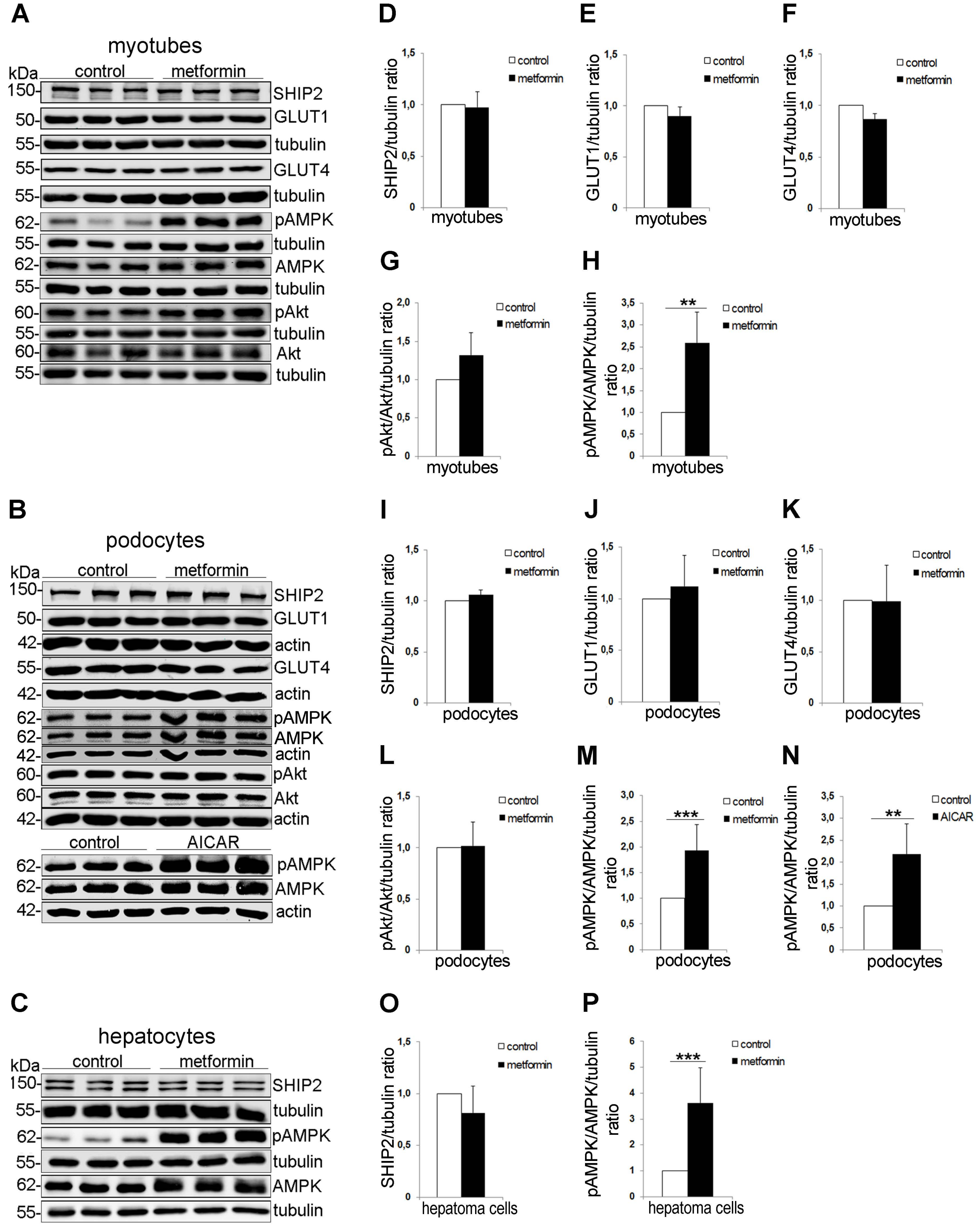

Supplemental Figure 4

A **B**

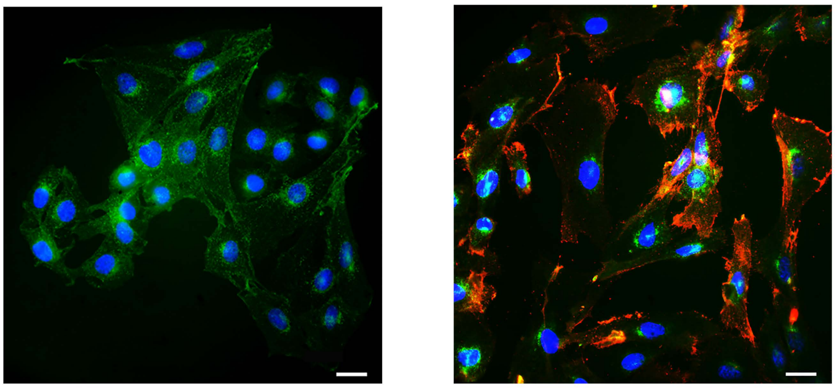

Supplemental Figure 5

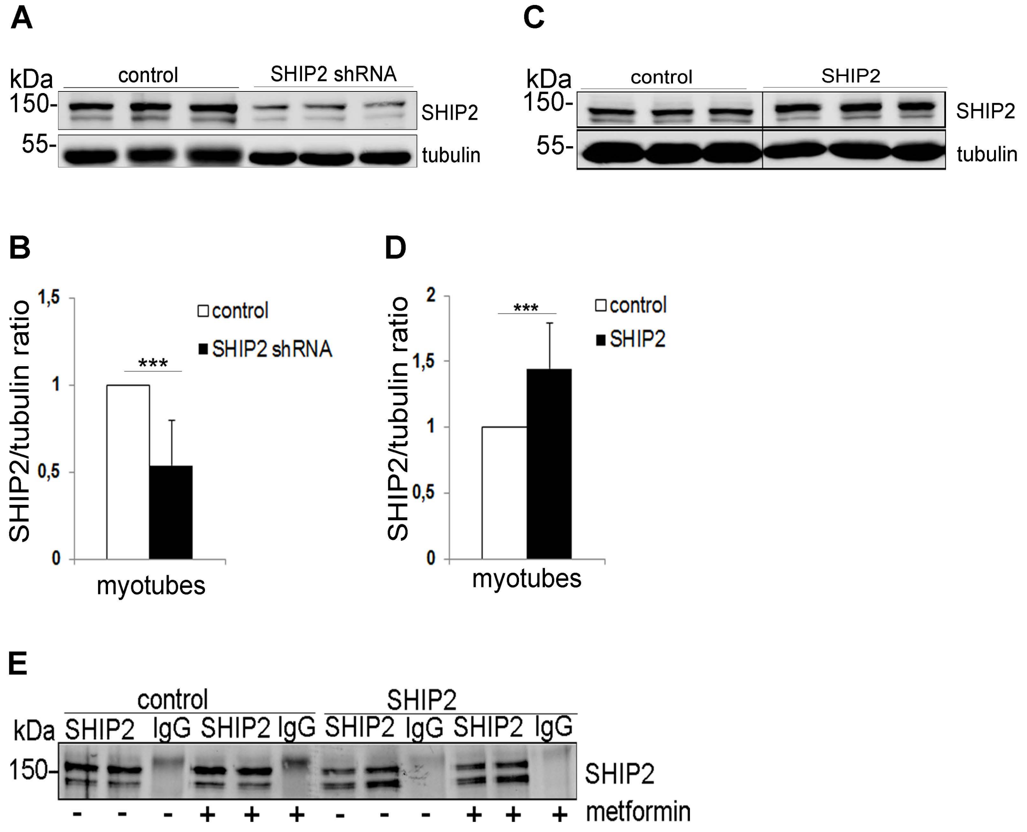

Supplemental Figure 6

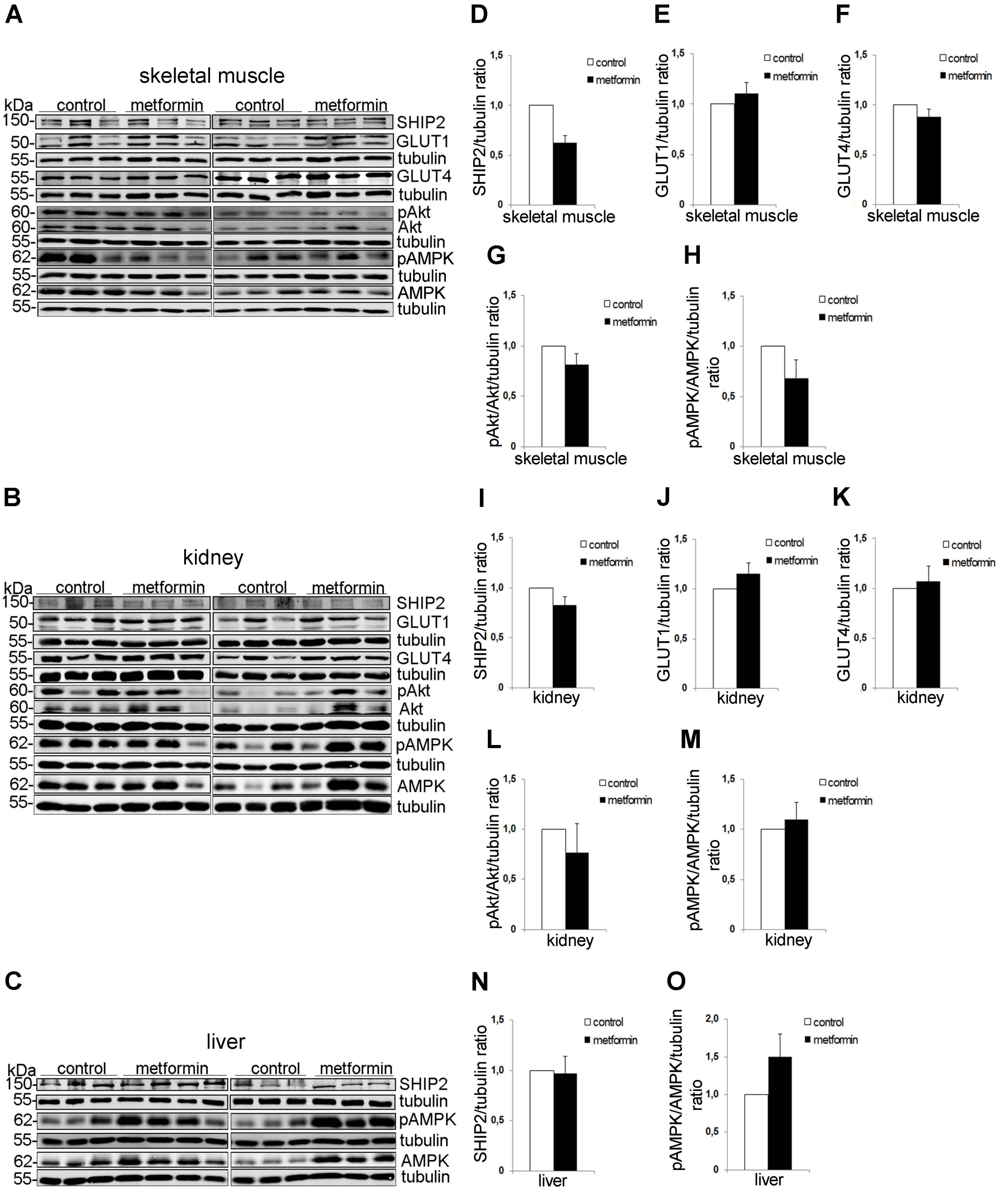

Supplement: Supplementary file 1 [file fj.201800529RR.sd1.pdf]
